# Bio-nanocoatings based on castor oil enhanced with nanomaterials as corrosion reducers in injection wells pipelines

**DOI:** 10.1039/d5na00317b

**Published:** 2025-08-06

**Authors:** Juan D. Quintero, Yurany Villada, Helen Iniciarte, Claudia Gomez, Esteban A. Taborda, Luis Rios, Camilo A. Franco, Farid B. Cortés

**Affiliations:** a Grupo de Investigación Fenómenos de Superficie-Michael Polanyi, Facultad de Minas, Universidad Nacional de Colombia Sede Medellín Kra 80 No. 65-223 Colombia caafrancoar@unal.edu.co fbcortes@unal.edu.co; b Grupo de Investigación Procesos Químicos Industriales, Universidad de Antioquia, Sede Investigación Universitaria – SIU Lab. 329 Torre 2 Medellín Colombia grupopqi@udea.edu.co

## Abstract

Corrosion is a recurring problem in the oil and gas industries. The application of coatings has been demonstrated to prevent the corrosion of pipelines and associated infrastructure, reducing maintenance and repair costs. In this study, an alkyd-urethane coating based on castor oil with the addition of alumina (Al_2_O_3_), carbon quantum dots (CQDs), and silica (SiO_2_) nanoparticles as corrosion reducers in injection-well pipelines is evaluated. The use of this bio-based resin combined with nanoparticles represents an innovative approach to develop sustainable anticorrosive coatings. Potentiodynamic polarization (ASTM 59–97) with and without CO_2_, electrochemical impedance spectroscopy and corrosion resistance tests were used to evaluate the effect of nanomaterials on the anticorrosive performance of the coatings. The effects on rheological properties were determined using steady and dynamic rheology. Furthermore, the changes in the microstructure coating were observed by scanning electron microscopy (SEM). Based on potentiodynamic analysis, the coating in the presence of nanoparticles increased the corrosion potential and reduced the corrosion rate. Notably, the coating with 100 mg per L CQDs exhibited the best performance with respect to corrosion potential and current corrosion with and without CO_2_. In particular, the efficiency of corrosion inhibition of the CQDs coating was 99.9%. However, the coating with 100 mg L^−1^ of Al_2_O_3_ showed better corrosion resistance over time to salt spray exposure and electrochemical impedance test. The resin exhibited Newtonian behaviour, with a viscosity of 150 cP at 25 °C. On the other hand, the resin exhibited viscoelastic behaviour with *G*′′ > *G*′ in the evaluated frequency range. The SEM results confirm the incorporation of nanoparticles resulting in structural changes of coating. Based on these results, nanomaterial enhanced castor oil-based coatings can be a promising alternative to inhibit the corrosion generated in injection wells and promote sustainability using renewable raw materials. This work advances the field of sustainable anticorrosive coatings, with potential applications extending beyond injection wells to marine, infrastructure, automotive, among others underscoring its broad industrial and environmental impact.

## Abstract 2

Corrosion is a recurring problem in the oil and gas industries.

## Graphical abstract

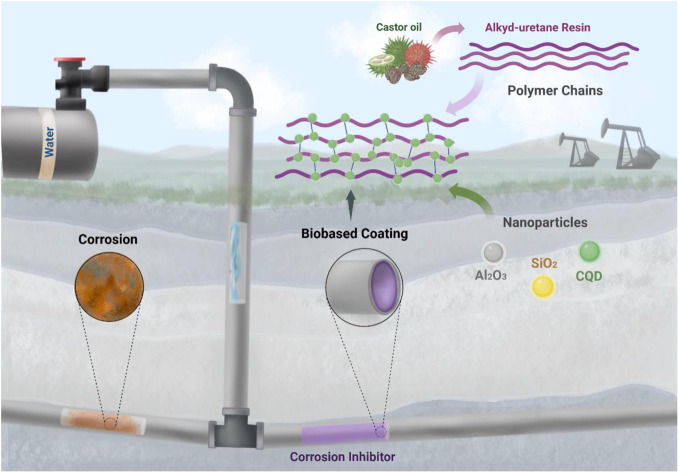

## 1. Introduction

A recurring problem in the oil and gas industry is the corrosion of structures involved in all stages of hydrocarbon exploitation.^1^ The oil and gas (O&A) industry spends about USD 60 billion per year on new construction, preventive strategies, mitigation interventions, and correction of failures related to corrosion and wear.^2,3^ Corrosion in the O&G industry occurs through several mechanisms such as electrochemical or chemical corrosion and mechanical effects.^4^ The presence of water in both injection and production wells, ionic species, sand, microbes, hydrochloric acid (HCl), carbon dioxide (CO_2_), sulfidic acid (H_2_S), and oxygen (O_2_) is the leading cause of corrosion.^5^ The reduction in pipe thickness promotes the loss of mechanical properties of the materials.^6,7^ The loss of mechanical properties, including resistance, ductility, and impact resistance, can lead to a range of issues, including leakage, rupture, and breakage in pipelines as well as environmental damage and economic losses.^8^ Additionally, because of the increase in pipe roughness, turbulence of the fluids and friction losses are promoted, increasing the energy consumption and the generation of carbon dioxide (CO_2_) emissions.^9^ Furthermore, corrosion increases operation and maintenance costs owing to equipment failure, production loss, and preventive programs.^10^ Consequently, interventions such as cleaning, pulling, workover operations, rig reconditioning, pump maintenance, and surface-line fault corrections have become more frequent.^11^

Several strategies for corrosion control have been proposed, including the use of inhibitors,^12^ resistant alloys,^13^ and coatings.^14^ The use of inhibitors based on amines, imidazolines, chromates, and polymers slows or inhibits corrosion.^15^ However, most inhibitor compounds have harmful effects on the environment, and are expensive and toxic.^16^ Recent advances in bio-based inhibitors have shown alternatives promissors, offering environmentally friendly advantages to traditional inhibitors.^17,18^ In particular, bio inhibitors derived from natural sources such as plant-based extracts have demonstrated enhanced corrosion protection through the formation of protective films and improved barrier properties.^19^ The extracts in some plants contain alkaloids, amino acids, polyphenols, that could form cyclic compounds to interact with metallic surfaces reducing the corrosion.^20^ Authors such as Bendaif *et al.*^21^ studied polyphenols from pancratium foetidum pom as corrosion inhibitors in HCl solutions at 1 M. They found inhibition efficiencies of 95% at 1 g L^−1^ inhibitor concentrations. Similarly, Kemel^22^ evaluated phenolic compounds from cynara syriaca as corrosion inhibitors in acidic media, finding inhibition efficiencies of 94% at low temperatures, and 73% at high temperatures. Furthermore, these compounds are ineffective against all types of corrosion and provide temporary or short-term protection. Alloys that are resistant to specific environments are also a strategy for preventing corrosion. Stainless steel alloys (316 L) are employed when corrosion resistance is crucial.^23^ Similarly, duplex stainless steels exhibit corrosion resistance and high strengths.^24^ However, high costs can be a significant factor in large-scale projects. Conversely, a common corrosion-prevention method used for metallic structures in water pipelines is the application of coatings.^25,26^ Coating reduces friction and interaction between the fluid and metal surface.^27^ Depending on their applications, coatings can be formulated using polymeric,^28^ ceramic,^29^ or metallic materials.^30^ When the coatings are applied correctly, they can protect over 99% of the surface area of pipelines.^31,32^ The most commonly used internal coating for injection well pipes is epoxy coating.^33,34^ Epoxy coatings form a smooth and impermeable barrier that protects the pipelines from corrosive fluids and minimizes the frictional resistance.^35^ Chen *et al.*^36^ studied the application of a fusion-bonded epoxy coating in one of the world's largest saltwater injection systems. The results showed that the coating improved the injection rate and decreased the iron content of water. However, the physicochemical properties of water are maintained to prevent coating damage.^37^ Another type of coating used for pipelines with a high risk of corrosion is the glass-reinforced epoxy. However, they are expensive and exhibit a low resistance to abrasion.^38,39^ On the other hand, epoxy coatings have been combined with polymers like polyethylene,^40^ polyamide,^41^ and polyurethane^42^ as an alternative method for enhancing adhesion and boosting resistance on pipelines.^43,44^ In this treatment, the first layer is an epoxy resin that generates good adhesion to the metal surfaces, thereby supporting the outer layers.^45^ The outer layers can be made of materials, such as polyethylene or polyurethane, which provide greater chemical resistance to the coating.^46^ However, 90% of these coatings are synthesized using bisphenol A (BPA), which is toxic and harmful to the environment as well as human health.^47^ Moreover, epoxy polymers contain polar groups, such as amine, hydroxyl, and epoxy groups, leading to water absorption and corrosion of the substrate metal.^48^ In addition, the availability and potential environmental impact of non-renewable resources for coating manufacturing highlight the need for coating precursors based on renewable sources.^49^ Hence, bio-based treatments have been proposed as a sustainable alternative based on vegetable oils such as castor oil and other green coatings.^50^ Among them, castor-oil-based coatings, including polyurethane,^51^ poly (ester amide),^52^ and alkyd,^53^ have been studied in recent years.^54^ Nevertheless, their anticorrosive application still has challenges, such as adhesion problems on different substrates, curing specific conditions, mechanical properties, and chemical resistance.

Nanotechnology as part of convergent technologies, offers a promising solution for this type of process.^55–57^ Due to their physical–chemical properties and their tiny size (<100 nm), nanoparticles can improve the performance of coatings.^58^ The incorporation of nanoparticles in the coatings has been used to enhance the thermal stability of the polymer, making it resistant to scratches and abrasion, and improving the corrosion resistance.^59,60^ Nanomaterials such as graphene and silica have been used to improve the thermal and chemical resistance of materials.^61^ Also, nanoparticles based on metal oxides such as alumina (Al_2_O_3_),^62^ cerium oxide (CeO_2_),^63^ zinc oxide (ZnO),^64^ copper oxide (CuO),^65^ and titanium oxide (TiO_2_)^66^ has been studied to improve the mechanical, thermal, electrical, and chemical resistance, antifouling, and durability.^67,68^ These oxides facilitate passivation of the surface, promoting the formation of a stable oxide layer that protects against additional corrosion.^69^ Similarly, ZnO nanoparticles are commonly used for corrosion protection.^70^ These nanoparticles improve the corrosion resistance through two mechanisms: barrier and cathodic protection.^71^ Alumina nanoparticles have been widely used to reinforce metal or polymer matrices because of their mechanical properties, high hardness, high thermal stability, and corrosion resistance.^72^ Chang *et al.*^73^ studied the reinforcement of polyurethane-type coatings with alumina nanoparticles. The results showed that nanoparticles adsorb resin, enhancing the coating cross-link density and decreasing the path through which corrosive electrolytes can permeate. Although significant progress has been made in reinforcing bio-based polyurethane and alkyd coatings with nanoparticles,^74,75^ the synergistic performance of hybrid alkyd-urethane coatings modified with nanoparticles remains largely unexplored. Furthermore, there is not publication related with alkyd-urethane coating obtained from castor oil enhanced with nanoparticles. In this sense, the developed durable, sustainable coatings tailored for such critical applications in oil and gas industries represent an attractive alternative.

The main objective of this study is to develop nanoparticle-modified alkyd-urethane coating, and its evaluation as corrosion inhibitor under injection parameters including a representative multicomponent brine and the presence of CO_2_. A castor-oil-based bio-nanocoating was enhanced using nanomaterials of alumina, CQDs and silica. This research includes: (i) evaluation of the coatings anticorrosive performance using electrochemical polarization, electrochemical impedance spectroscopy, corrosion resistance, and CO_2_ bubble test; (ii) the effect of nanoparticles in the rheological properties of the coating through steady and dynamic rheology; and (iv) effect of nanomaterials in the structural properties of coating by SEM. This study establishes the promising potential of alkyd-urethane coatings for injector well corrosion protection as an environmentally alternative to conventional coatings. Besides, the research implements the use of nanotechnology to improve the functional properties of the coatings. To evaluate the synergistic effects of different nanomaterials with an alkyd-urethane coating, three nanoparticles based on their distinct chemical nature were selected. Al_2_O_3_ nanoparticles due to their high mechanical strength, chemical stability, and ability to form dense protective barriers, which are especially beneficial in high-salinity and high-temperature environments.^76,77^ CQDs, as carbon-based nanoparticles due to their capacity to enhance corrosion inhibition through the formation of uniform and adherent protective films on metal surfaces.^78,79^ SiO_2_ due to its chemical inertness and its wide use to improving the structural integrity, dispersion stability, and barrier properties of coatings.^80,81^

## 2. Materials and methods

### 2.1 Materials

Alkyd-urethane resins were synthesized from a dehydrated castor oil alkyd resin obtained from the Grupo de Investigación Procesos Químicos Industriales at Universidad de Antioquia facilities (Medellín, Colombia). The isophorone diisocyanate and cobalt octoate used for the synthesis were purchased from Sigma-Aldrich (Saint Louis, USA). The primer employed to enhance adherence was obtained from the MTN Colors Company (Barcelona, Spain). Brine was prepared using sodium chloride (NaCl), sodium sulfate (Na_2_SO_4_) and sodium bicarbonate (NaHCO_3_) from Honeywell (Charlotte, USA). Hydrochloric acid (HCl) and isopropyl alcohol used for the metal surface cleaning were purchased from Merck Millipore (Darmstadt, Germany) and Protokimica S.A.S. (Medellín, Colombia), respectively. The citric acid and the ethylenediamine used for the synthesis of carbon quantum dots (CQDs) were obtained from Honeywell (Charlotte, USA) and Merck Millipore (Darmstadt, Germany), respectively. Carbon dioxide (CO_2_) used in the bubble test was provided by Cryogas (Medellín, Colombia). All reagents were used as received without further purification. Carbon steel coupons were provided by Laminas y Cortes S.A.S (Medellin, Colombia) and used for coatings application. To evaluate the impact of nanoparticles on the coating performance, CQDs, alumina (Al_2_O_3_) and silica (SiO_2_) nanoparticles were considered. The CQDs were synthesized according to the procedure described by Franco *et al.*^82^ through microwave-assisted synthesis. The obtained CQDs have a mean hydrodynamic diameter of 30 nm.^82^ Al_2_O_3_ and SiO_2_ nanoparticles were provided by Petroraza SAS (Medellín, Colombia) and Sigma-Aldrich (Saint Louis, USA), respectively. Each type of nanoparticle has a mean hydrodynamic diameter of 35 nm and 11 nm. The sizes reported correspond to hydrodynamic diameter measured with Dynamic Light Scattering (DLS) technique. Al_2_O_3_ and SiO_2_ have BET surface area values of 123 m^2^ g^−1^ and 210 m^2^ g^−1^, respectively. The surface area of CQDs was estimated using TEM image analysis, obtaining a value of 121 m^2^ g^−1^. Additional information regarding nanoparticles characterization can be found in previous studies.^83,84^

### 2.2 Synthesis of the coating

The alkyd-urethane resin was synthesized in a Petri dish following the procedure proposed by Villada *et al.*^85^ To this end, 5 g of dehydrated castor oil alkyd resin was stirred manually at room temperature for 5 min. Then, 1.59 g of isophorone diisocyanate was added and stirred at room temperature for 5 min. Further, 1.32 g of acetone was added, and the mixture was stirred for 5 min. Finally, 0.03 g of cobalt octoate was added as the drying agent. The properties and additional characterization of the obtained resin can be found in Table 1 according to a previous study.^85^

**Table 1:** Physicochemical properties of alkyd-urethane resin. Adapted from Villada *et al.*^85^

Property	Alkyl urethane resin
Chemical resistance	Water, H_2_SO_4_^a^, NaOH^b^^,^^c^, acetone^c^, xylene^c^
Gloss values	20°: 90.1, 35°: 84.3, 60°: 120.1
Pencil hardness	Gouge hardness: 3H, scratch hardness: 4H
Thermal stability	220 °C

a Unaffected.

b Film swells.

c Film slightly removed.

### 2.3 Coating formation

Carbon steel coupons of 2 × 7 cm were sanded, treated with HCl to remove surface oxides, and cleaned with acetone and isopropyl alcohol to eliminate contaminants, such as grease. A brush was used and dipped into the coating and excess paint was tapped off. The coating was then applied with smooth movement while maintaining a wet edge to prevent brush marks and dried in an oven (ThermoFisher Scientific, USA) at 100 °C for 1 h. The film thickness was determined using a coating tester (CEM DT-156, CEM Instruments, China). The performance of the coatings was tested in a characteristic brine (NaCl: 23.38 g L^−1^, Na_2_SO_4_: 3.41 g L^−1^, NaHCO_3_: 0.170 g L^−1^) at 70 °C. It is important to note that before the resin was applied to the coupon, an inner layer of primer was applied to increase coating adherence. Different coatings were prepared to evaluate their performance as corrosion reducers in the injection pipelines as it presented in Table 2. To assess the effect of several nanoparticles on the performance of the coatings, alumina, CQDs, and silica nanoparticles were added during the synthesis process. Specifically, concentrations of 10, 100, and 1000 mg L^−1^ of the nanoparticles were mixed with acetone and sonicated for 1 h to achieve a well-dispersed mixture and disrupt nanoparticle clusters. This mixture was used in the synthesis process in the stage of the solvent addition. In this study, the uncoated coupon served as the blank, while the coupon coated without nanoparticle was designated as the base.

**Table 2:** Systems studied

System	Description
Blank	Uncoated coupon
Base	Coupon coated with alkyd-urethane resin
Base + Al_2_O_3_	Coupon coated with alkyd-urethane resin with 10, 100, or 1000 mg L^−1^ of the nanoparticles
Base + CQDs	
Base + SiO_2_	

### 2.4 Coating performance

#### 2.4.1 Polarization test

Electrochemical tests were performed using a Gamry Interface 1000 potentiostat (Gamry instruments, USA) to evaluate the anticorrosive performances of the coatings. A 3-electrode assembly was made following the ASTM G59–91 standard,^86^ in which the metal-coated coupon acted as the working electrode. A graphite counter electrode was used to close the circuit and allow current flow, and an electrode of silver/silver chloride (Ag/AgCl) was used as a reference. A linear polarization resistance test was conducted with a potential difference scan between −0.25–0.25 V, a scan speed of 0.2 mV s^−1^, and the current flow response through the system was observed. The corrosion potential and the corrosion current were obtained from the Gamry Echem Analyst 2 (Gamry instruments, USA) and the efficiency of corrosion inhibition of the coating was calculated using eqn (1).1
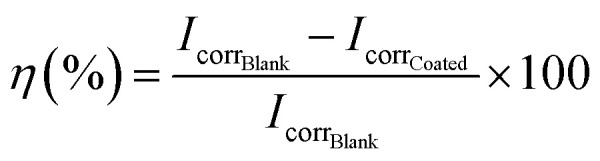
where *I*_corrBlank_ is the corrosion current of the blank coupon and *I*_corrCoated_ is the corrosion current of the coated coupon.

#### 2.4.2 Electrochemical impedance spectroscopy

An electrochemical impedance spectroscopy (EIS) analysis was performed using a three-electrode cell. An alternating current (AC) signal was applied in the frequency domain, ranging from 100 kHz to 10 MHz with an amplitude of 10 mV, in a brine solution. The amplitude of the sine wave is ±10 mV. Polarization resistance (*R*_p_) was evaluated after 1, 5, and 20 days of brine immersion. *R*_p_ values were determined by fitting electrochemical impedance using the Gamry Echem Analyst 2 (Gamry instruments, USA).

#### 2.4.3 CO_2_ bubble test

To evaluate the coating performance in the presence of CO_2_ in the brine, a linear polarization test was performed. To saturate the brine, CO_2_ was bubbled for one hour at a flow rate of 6 L min^−1^ at atmospheric pressure. The test was conducted with a potential difference scan between −0.25–0.25 V, a scan speed of 0.2 mV s^−1^, and the current flow response through the system was observed.

#### 2.4.4 Corrosion resistance test

The corrosion resistance was evaluated by exposing coated coupons to a 60 °C brine spray within a sealed chamber in an oven (ThermoFisher Scientific, USA). The spray was applied at 30-minute intervals for 6 hours, and the corrosion progress was subsequently observed. The visual evaluation was conducted following ASTM D1654 standard. Particularly, the scribe creep was estimated and a numerical rating from 0 (complete failure) to 10 (no visible creep or rust), was assigned. This semi-quantitative rating allowed for comparative analysis of the corrosion protection offered by the coatings.

### 2.5 Rheological characterization of the resin

The rheology measurements were carried out using a Kinexus-Pro rheometer (Malvern, USA) with a cone-plate geometry at 25 °C. Steady shear measurements were performed at shear rates of 0 and 100 s^−1^ at room temperature. Likewise, the frequency sweep tests were performed from 0.01 to 10 Hz within the linear viscoelastic region at strains of 0.5 and room temperature. The linear viscoelastic region was determined using strain sweep tests from 0.01 to 100 at 10 Hz. The test was repeated twice.

### 2.6 Scanning electron microscopy (SEM)

Scanning electron microscopy (SEM) images were recorded using a JEOL JSM-7100 microscope (JEOL, Japan) equipped with a field emission gun (FEG) and an auxiliary detector for retro scattered electrons operating at an acceleration voltage of 15 kV. For this assay, three coatings were selected: an alkyd-urethane coating (base) and coatings modified with alumina nanoparticles and CQDs. The mean diameter of nanoparticles was quantified using ImageJ software (version 1.53). Aggregates were identified as distinct clusters of nanoparticles with a contrast threshold set to differentiate them from the polymer matrix. The diameter of each aggregate was calculated by setting a bar scale with the SEM reference, and the mean diameter was determined by averaging across all detected aggregates.

### 2.7. Surface interactions

Fourier Transform Infrared (FTIR) spectroscopy was employed to confirm and identify the interactions of nanoparticles with the alkyd-urethane coating. Samples of the cured coatings were prepared by maceration and subsequent homogeneous mixing with spectroscopic-grade potassium bromide (KBr). The test was carried out using an IRAffinity-1S infrared spectrophotometer (Shimadzu, Kyoto, Japan). The measurement was performed at room temperature in the wavenumber range of 4500–650 cm^−1^ with a resolution of 2 cm^−1^. The resulting spectra were then analyzed using Shimadzu LabSolutions IR software, performing baseline correction and identifying characteristic absorption bands.

## 3. Results

### 3.1 Coating performance

#### 3.1.1 Polarization test

**Fig. 1:**
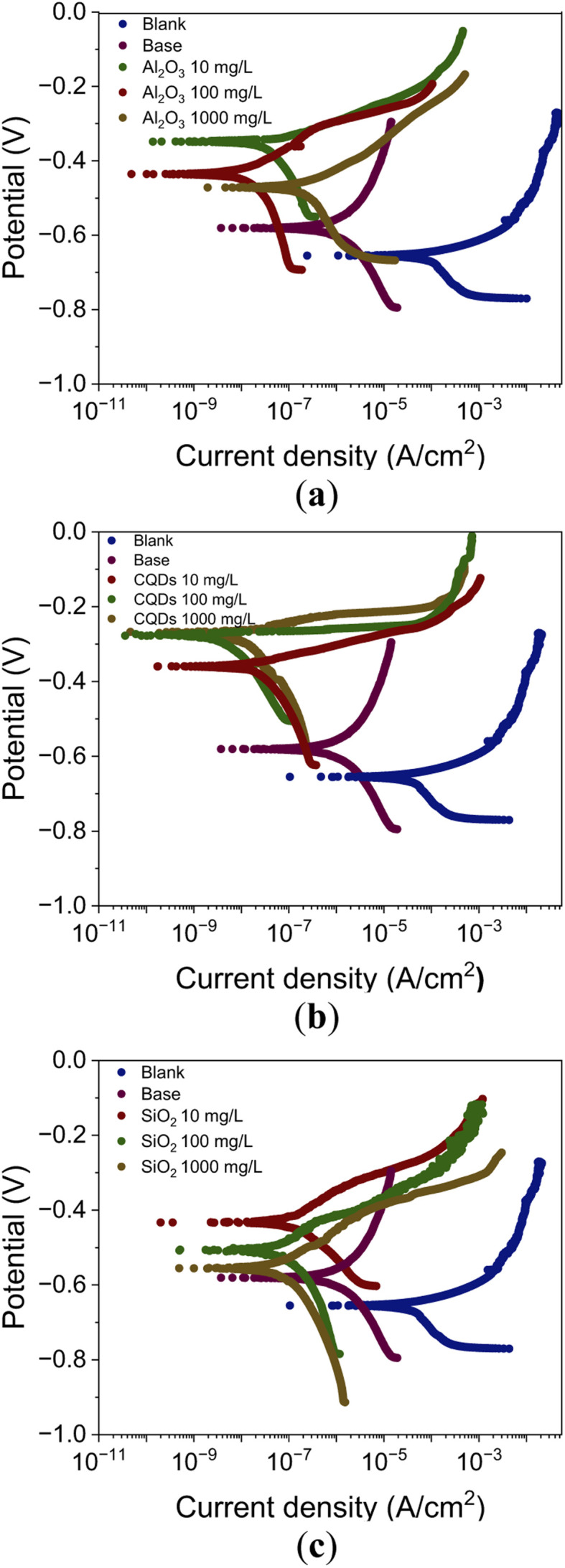
Polarization curves of the blank (uncoated coupon), the base coating, and the coating with 10, 100 and 1000 mg L^−1^ of (a) alumina, (b) CQDs, and (c) silica nanoparticles. The tests were carried out in a 3-electrode setup with reference (Ag/AgCl) and graphite as counter electrode at 25 °C and atmospheric pressure.

The effects of the addition of nanoparticles at several concentrations on the electrochemical performance of the coatings are shown in Fig. 1 and Table 3. It can be observed that alkyd-urethane coating (base) increases the corrosion potential from −0.65 to −0.60 V and decreases the corrosion current from 5.940 × 10^−5^ to 0.088 × 10^−5^ A cm^−2^ compared with the blank coupon (uncoated coupon). The shift towards a less negative corrosion potential in the coated sample suggests a decrease in the thermodynamic spontaneity of metal oxidation reaction, demonstrating the coatings effectiveness in reducing corrosion.^87^ The spontaneity of electrochemical reactions is governed by the Gibbs free energy expressed as Δ*G* = −*nFE* where *n* is the number of electrons transferred, *F* is the Faraday constant, and *E* is electrochemical potential. A less negative *E*_corr reduces the driving force for corrosion generating the oxidation reaction less favorable. Conversely, the decrease in the current means that the corrosion reaction is slowing, indicating a reduction in the electrochemical activity at the surface and suggesting the formation of the protective layer.^88^ It is observed in Fig. 1a that coatings with 10, 100, and 1000 mg L^−1^ of alumina nanoparticles increase the corrosion potential from −0.60 V to −0.35, −0.44 and −0.45 V, respectively. Likewise, the corrosion current of the coating with nanoparticles decreased from 0.088 × 10^−5^ to 0.006 × 10^−5^, 0.001 × 10^−5^, and 0.015 × 10^−5^ A cm^−2^, respectively. Fig. 1b shows the effect of the addition of several concentrations of CQDs on the electrochemical performance of the coatings. The results indicated that the CQDs increased the corrosion potential and decreased the corrosion current at all the concentrations evaluated. The corrosion potential of the coating with 10, 100, and 1000 mg L^−1^ of CQDs increases from −0.60 to −0.36, −0.28, and −0.30 V and the corrosion current decreased from 0.088 × 10^−5^ to 0.0048 × 10^−5^, 0.0009 × 10^−5^, 0.0012 × 10^−5^ A cm^−2^, respectively. Furthermore, the effect of adding silica nanoparticles on the alkyd-urethane coating is shown in Fig. 1c. The Tafel curves reveal that the addition of 10, 100, and 1000 mg L^−1^ concentrations of silica nanoparticles increases the corrosion potential from −0.60 to −0.42, −0.50, and −0.57 V and decreases the corrosion current from 0.088 × 10^−5^ to 0.0085 × 10^−5^, 0.0056 × 10^−5^ and 0.0086 × 10^−5^ A cm^−2^, respectively.

**Table 3:** Electrochemical parameters obtained from the polarization test

Coupon	Concentration	*E*_corr_ ± 0.01 (V)	*I*_corr_ (×10^−5^) ± 1 × 10^−9^ A cm^−2^	Corrosion rate (×10^−2^) ± 1 × 10^−5^ mpy	*η* ± 0.1 (%)
Blank	—	−0.65	5.8921	3.306	—
Base	—	−0.60	0.0882	0.051	98.5
Al_2_O_3_	10	−0.35	0.0057	0.002	99.9
	100	−0.43	0.0013	0.001	99.9
	1000	−0.47	0.0151	0.004	99.4
CQDs	10	−0.36	0.0048	0.001	99.9
	100	−0.28	0.0009	0.001	99.9
	1000	−0.27	0.0012	0.001	99.9
SiO_2_	10	−0.42	0.0085	0.003	99.8
	100	−0.50	0.0056	0.002	99.9
	1000	−0.57	0.0086	0.003	99.8

Table 3 shows the electrochemical parameters obtained from the polarization test. It can be observed that the corrosion rates for the blank and the base coating were 3.3 × 10^−2^ and 0.05 × 10^−2^ mpy, respectively. This result indicates the effectiveness of the base coating. For coatings with alumina nanoparticles at 10, 100, and 1000 mg L^−1^ the corrosion rates were 0.002 × 10^−2^, 0.001 × 10^−2^, and 0.004 × 10^−2^ mpy, respectively. Compared to the blank the inhibition efficiencies of these coatings were 99.9, 99.9, and 99.4%, respectively. On the other hand, the coatings with CQDs nanoparticles present corrosion rates and inhibition efficiency of 0.001 mpy and 99.9%. Note that, the varying CQD concentration does not produce a significant effect on the electrochemical parameters. Regarding the coatings with 10, 100, and 1000 mg L^−1^ of silica nanoparticles, the corrosion rates were 0.003, 0.002, and 0.003 mpy while the corrosion inhibition efficiencies were 99.8, 99.9, and 99.8%, respectively. The decrease current densities values leads to the increase in the surface coverage values.^18^ However, aggregates formation may reduce surface coverage at high concentrations. The anodic slopes become less steep, and the anodic current densities are lower in coatings with nanoparticles, compared to resin without them. This suggests that metal oxidation is being inhibited and the nanoparticles, as a barrier, limit the access of corrosive agents to the metal.^17,19^ Therefore, it can be concluded that the coating enhanced with nanomaterials acts as a barrier that impedes the current flow between the electrode and electrolyte, preventing the occurrence of oxidation reactions in the metal surface. These results may be associated with the urethane groups promoting the formation of a dense and compact three-dimensional network in the coating that prevents the penetration of corrosive agents.^89^ Additionally, the presence of nanoparticles enhances the anticorrosive properties of the coating due to the different physicochemical interaction mechanisms between the functional groups of the coating and the nanoparticles. Several researchers have concluded that the nanoparticles can interact with coatings through different mechanisms, including van der Waals forces, electrostatic forces, hydrogen bonds, ionic interactions, and steric interactions.^90^ In particular, van der Waals forces are fundamental to the attraction between polymers and nanoparticles.^91^ Although the van der Waals forces are weak, their effects contribute to the adhesion between the polymer and the nanoparticles. Besides, polymers and nanoparticles can carry charges on their surfaces owing to the presence of ionizable functional groups.^92^ If the charges are opposite, an attractive force is generated, causing the polymer and the nanoparticle to be closer.

Furthermore, the presence of functional groups such as hydroxyl, carbonyl, and amine groups present in the polymer and the nanoparticles promotes hydrogen bond formation.^93^ This type of bonding is stronger than van der Waals forces but weaker than covalent bonds, significantly affecting the coating properties. In addition, depending on the functional groups of the polymer and nanoparticles, they can interact through ionic bonds.^94^ On the other hand, the polymers can act as steric barriers that prevent the aggregation of nanoparticles, keeping them dispersed in the matrix.^95^ Specifically, urethanes (–NHCOO–) and isocyanate groups (–NCO) present in the alkyd-urethane coating can interact with nanoparticles through hydrogen bonds and electrostatic forces.^96^ Likewise, the nanoparticles strengthen the polymer matrix of the coating, preventing the formation of cracks that caused corrosion. The large surface-to-volume ratio of nanoparticles enhances their interaction with polymers, leading to the formation of an nanoparticle–polymer interface that modifies the physical properties of the surrounding polymer.^97^ Well-integrated nanoparticles can act as physical crosslinking points, increasing the cohesive energy density of the polymer network.^98^ Moreover, interfacial interactions can influence the thermal and oxidative stability of the coating. Specifically, Al_2_O_3_ and SiO_2_ nanoparticles can interact with the polymer to form thermally stable surfaces that retard thermal degradation.^99^ Similarly, carbon-based CQDs, can enhance UV resistance and inhibit oxidative reactions that degrade the polymer over time due to their high surface area and functional groups.^100^ However, higher concentrations can promote clustering, reducing the available surface area of nanoparticles, and weakening the barrier effect.^101^ Therefore, this could disrupt the tortuosity effect or weaken the interaction with the coating, compromising overall effectiveness. These results are in agreement with Dandan Doganci and Sevinç,^102^ Hosseinpour *et al.*,^103^ and Sharma *et al.*^104^ who found that alumina, CQDs, and silica nanoparticles increase the performance of organic coatings in polarization tests. Particularly, the surface of alumina nanoparticles can contain hydroxyl (OH) groups that can form hydrogen bonds with the carbonyl (C=O) groups of urethane bonds.^105^ Similarly, the results obtained with the addition of the CQDs can be attributed to the chemical interactions between them and the organic coating. The corrosion resistance of the CQD coating could be attributed to the potential for hydrogen bonding between the CQDs and organic matrix.^106^ In addition, CQDs have a graphene SP^2^ core, allowing π–π interactions with the π bonds of the polymers, especially in regions of high electronic density.^107^ Moreover, CQDs can act as electron donors or acceptors, leading to the formation of charge-transfer complexes with the functional groups of the coating.^108^ However, the hydrophilicity of CQDs promotes water absorption that can affect the coating efficiency.^109^ In contrast, silica nanoparticles contain silanol groups (Si–OH) that are highly reactive and can form hydrogen bonds with the carbonyl groups (C=O) of urethane and alkyd bonds.^110^ Nanoparticles, due to their high surface-to-volume ratio, significantly increase the effective interfacial area between the polymer composite and the pipeline surface.^111^ Moreover, nanoparticles functional groups can form bonds with the polymer chains and the hydroxyl or oxides groups on the pipeline surface potentially forming additional bonding points.^112^ Furthermore, nanoparticles can enhance electrostatic attraction to charged sites on the pipeline surface, improving initial adhesion.^113^

While Tafel polarization curves offer valuable information about the instantaneous corrosion rate of the coatings evaluated, they provide limited insight into the evolution of the coatings protective properties over time. To study the long-term stability and degradation mechanisms of the coating, electrochemical impedance spectroscopy (EIS) measurements were performed.

#### 3.1.2 Electrochemical impedance test

**Fig. 2:**
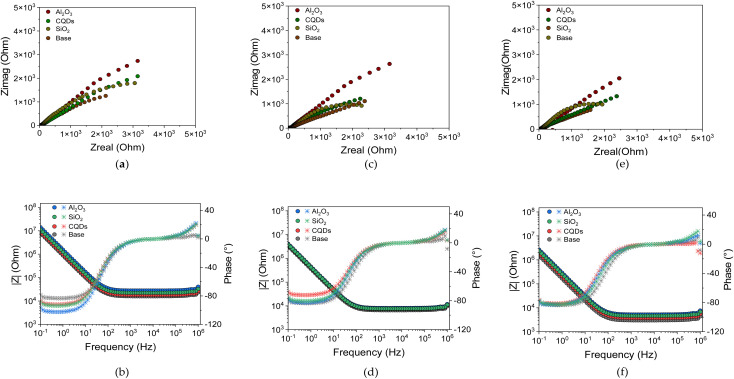
Nyquist and Bode curves for the electrochemical impedance spectroscopy test of the coated coupons with alkyd-urethane resin and 100 mg L^−1^ of the nanoparticles. Nyquist plot during (a) Day 0, (c) Day 5, and (e) Day 20, and Bode curves of the coated coatings during (b) Day 0, (d) Day 5, and (f) Day 20. The tests were carried out in a 3-electrode setup with reference (Ag/AgCl) and graphite as counter electrodes at 25 °C and atmospheric pressure.

As the best results in the polarization test were achieved with coatings containing 100 mg L^−1^ of nanoparticles, the electrochemical impedance evaluation was conducted using coatings with the same nanoparticle concentration. Fig. 2 shows the Nyquist and Bode curves for the electrochemical impedance spectroscopy test of the coatings after 1, 5 and 20 days of immersion in brine. The impedances of the base coating during days 1, 5, and 20 of exposition are shown in Fig. 2a, c, and e, respectively. The base coating impedance decreases from 1.1 × 10^3^ Ω to 0.8 × 10^3^ Ω in 20 days. The Nyquist curves for day 0 (Fig. 2a) show that the highest impedance was exhibited by the coating with 100 mg L^−1^ of alumina, reaching 3.2 × 10^3^ Ω. In contrast, the coatings with CQDs, SiO_2_, and base coatings showed impedances of 3.0 × 10^3^, 3.1 × 10^3^, and 2.2 × 10^3^ Ω, respectively. The Bode curves during day 0 of the exposure are shown in Fig. 2b. At low frequencies, the impedance modules show high values that decreased with higher frequencies. Besides, the impedance modules are higher for all the systems with nanoparticles addition. In Fig. 2c the Nyquist curves for coatings after 5 days are presented. The highest impedance was observed for the Al_2_O_3_ coating with an impedance of 3.3 × 10^3^ Ω. By contrast, the CQDs, SiO_2,_ and the base coatings evaluated show impedances of 2.3 × 10^3^, 2.4 × 10^3^ and 2.1 × 10^3^ Ω, respectively. After 5 days of immersion in brine, the Bode curves depicted in Fig. 2d are obtained. It is observed that the impedance modules decrease with high frequencies. Besides, the coating with nanoparticles shows slightly higher values of impedance than the base coating. The Nyquist curves after 20 days of exposure are shown in Fig. 2e. The highest impedance was observed for Al_2_O_3_ with a value of 2.5 × 10^3^ Ω. On the contrary, the CQDs, SiO_2_, and the base coatings evaluated show impedances of 2.0 × 10^3^, 2.0 × 10^3^ and 1.7 × 10^3^ Ω, respectively. The Bode curves after 20 days of exposure are shown in Fig. 2d. At low frequencies the impedance modules are higher in the coatings with nanoparticles. However, at high frequencies the coating with 100 mg L^−1^ of CQDs shows slightly lower values.

Overall, all coatings showed a higher impedance for the first day, indicating that they initially provided an effective barrier against corrosion. However, this behavior is more noticeable for the coating based on alumina. By the fifth day, a reduction in the impedance was noted across all cases, suggesting that the coating might deteriorate and allow increased electrolyte permeability to the metal surface. These results enable the acquisition of data that can be utilized to forecast the coating configuration, which demonstrates the least degree of degradation over time.^114^ Among the coatings with nanoparticles, the largest reduction in impedance was observed for those with silica and CQDs nanoparticles, indicating a higher reduction in the protection. Regarding the twentieth day, a decrease in impedance was noticeable in all coatings, revealing a higher permeability and water absorption. However, coatings with alumina maintain a higher impedance than the others, suggesting that they provide better long-term corrosion protection. The decrease in the impedance could be associated to the brine partially dissolved or degraded the organic layer, creating pathways for ions to flow more efficiently.^115^ In addition, absorbing water can lead to an increase in conductivity and a decrease in impedance.^116^ Thus, coatings containing water-soluble components like CQDs absorb water *via* osmosis, driven by differences in the salt concentration, explaining the CQDs coating impedance decay. This behavior led to a decrease in the ability of the coating to resist current flow, resulting in lower impedance.^117^ Similarly, the silica nanoparticles can promote the same effect.^118^

**Fig. 3:**
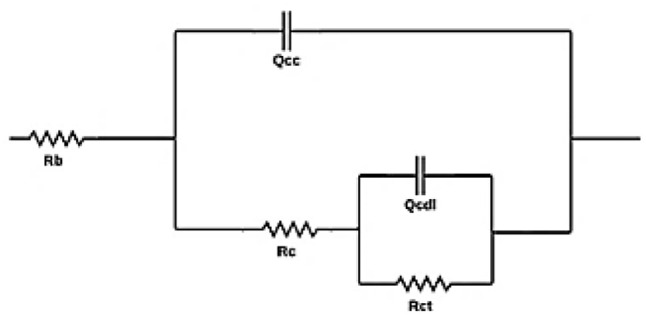
Equivalent circuit used as a model for the calculus of polarization resistance. This circuit includes the brine.

The Bode plots provide further insight into the impedance magnitude (|*Z*|) and phase angle as a function of frequency. At low frequencies, higher |*Z*| values indicate better corrosion resistance. The coating containing alumina nanoparticles shows higher |*Z*| values than the other coatings over time, confirming their enhanced protective behaviour. As the frequency increases, the impedance modulus decreases for all systems. This decrease is expected and related to the capacitive response of the coating. On the other hand, at low frequencies the phase angle approaches 80° for all systems, indicating a dominant capacitive behaviour. This is a characteristic of coatings that act as protective barriers. At intermediate frequencies, a slight variation in the phase angle is observed between the different systems. This could be related to structural differences generated by interactions with the nanoparticles. Fig. 3 depicts the equivalent circuit used as a model for the calculus of polarization resistance. This is a common model for analysing EIS data of polymer coatings on metallic surfaces. It can be represented as *R*_b_ + [*Q*_cc_∥(*R*_c_ + (*Q*_cdl_∥*R*_ct_))]. *R*_b_ represents the ohmic resistance of the electrolyte solution and any bulk resistance of the coating itself. *Q*_cc_ represents the non-ideal capacitive behaviour of the coating. A CPE (constant phase element) is used instead of a pure capacitor to account for surface roughness, porosity, and non-uniform current distribution. *R*_c_ is the resistance of the ionic path through pores or defects in the coating. The non-ideal capacitive behaviour of the electrochemical double layer formed at the metal electrolyte interface where *c* is represented by *Q*_cdl_. The resistance to charge transfer reactions at the metal-electrolyte interface is represented by *R*_ct_.

The polarization resistance (*R*_p_) values obtained from the EIS measurements with the equivalent circuit are listed in Table 4. Initially, after 1 day of exposure, all coated samples exhibited higher *R*_p_ than the base coating (15.3 kΩ). However, this effect is more noticeable for coating with Al_2_O_3_ showing the highest value of 23.1 kΩ, indicating their superior initial protective properties. On the contrary, the SiO_2_ and CQDs coatings achieved *R*_p_ around of 20.8 kΩ, and 16.9 kΩ, respectively. Over time, the base coating experienced a rapid decrease in *R*_p_ from 5.2 kΩ on day 5 to 2.5 kΩ on day 20, indicating high corrosion. In contrast, coating with Al_2_O_3_ nanoparticles maintains the highest resistance of 16.0 kΩ and 12.8 kΩ for the 5 and 20 days, respectively. The results suggest sustained protection of the coating. Coating based on SiO_2_ also presents potential anticorrosive performance but shows a faster decline of 11.3 kΩ at Day 5 and 6.7 kΩ at Day 20. Regarding the coatings CQDs, they offer moderate protection with a *R*_p_ of 7.3 kΩ at Day 5, and 4.5 kΩ after 20 days of exposure. These results align with the Nyquist and Bode curves, confirming that coating with Al_2_O_3_ provides the most durable corrosion resistance, followed by the coating with SiO_2_. Contrary, coating with CQDs offers intermediate protection, and the base coating degrades the fastest.

**Table 4:** Polarization resistance of the coatings evaluated with several nanoparticles at 100 mg L^−1^

Formulation	Day 0 (*R*_p_) ± 0.1 kΩ	Day 5 (*R*_p_) ± 0.1 kΩ	Day 20 (*R*_p_) ± 0.1 kΩ	Decay percentage ± 0.1 (%)
Base	15.3	5.2	2.5	83.6
Base + Al_2_O_3_	23.1	16.0	12.8	44.6
Base + CQDs	16.9	7.3	4.5	73.4
Base + SiO_2_	20.8	11.3	6.7	67.8

**Fig. 4:**
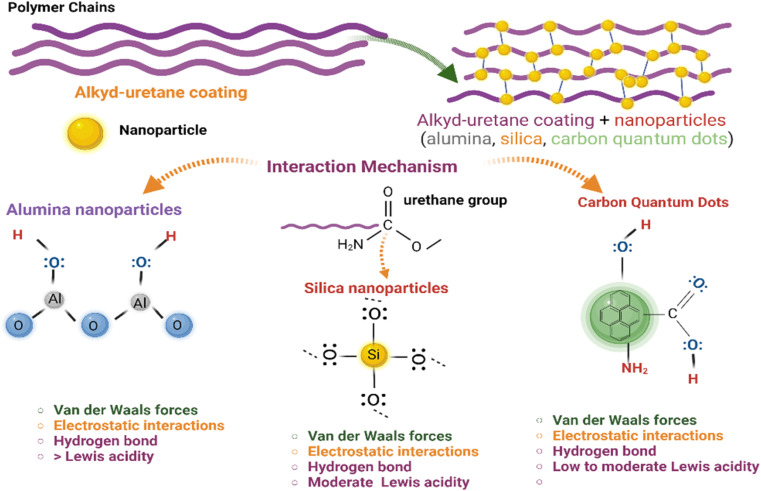
Schematic representation for the interactions between alkyd-urethane resin and alumina, CQDs, or silica nanoparticles.

Considering the several interactions previously mentioned, Fig. 4 schematically shows the possible interactions between the alkyd-urethane coatings and alumina, CQDs, or silica nanoparticles. The main functional groups for alumina are hydroxyl and epoxy. The silica contains silanol, and siloxane groups. On the other hand, CQDs have hydroxyl, carboxylic, carbonyl, and amine groups in their structure. For this reason, they can form several interactions, including the van der Waals forces, electrostatic interactions, and hydrogen bonds, among others. However, it is important to the analysis of results consider the Lewis acidity and the impedance of each nanomaterial. In particular, the alumina exhibits high Lewis acidity due to the incompletely coordinated Al^3+^ sites acting as strong electron acceptors.^119^ Likewise, alumina has a high impedance associated with its dielectric character, promoting high resistance to charge transfer and low electronic conductivity. The silica nanoparticles have moderate Lewis acidity related to the number of defects and Si–OH bonds on the surface. They also have an intermittent impedance due to electrical behavior that varies with hydration and surface modification.^120^ The CQDs potential has low Lewis acidity due to the few active sites present in their pure state. However, this acidity can be modified by the presence of nitrogen, oxygen, and boron. Also, they have low to moderate impedance associated with the conductive property of the material promoting high charge transfer.^121^ Based on this hypothesis, the results obtained with the polarization test showed that the CQDs and alumina coating have the best electrochemical performance against corrosion. However, these results couldn't be explained to the Lewis acidity and impedance. In this sense, the results are based on the several functional groups of CQD and alumina, and the particle size. As previously mentioned, the silica nanoparticles have a particle size of 11 nm, promoting low barrier effect and steric impediment in the polymer matrix.

#### 3.1.3 CO_2_ bubble test

**Fig. 5:**
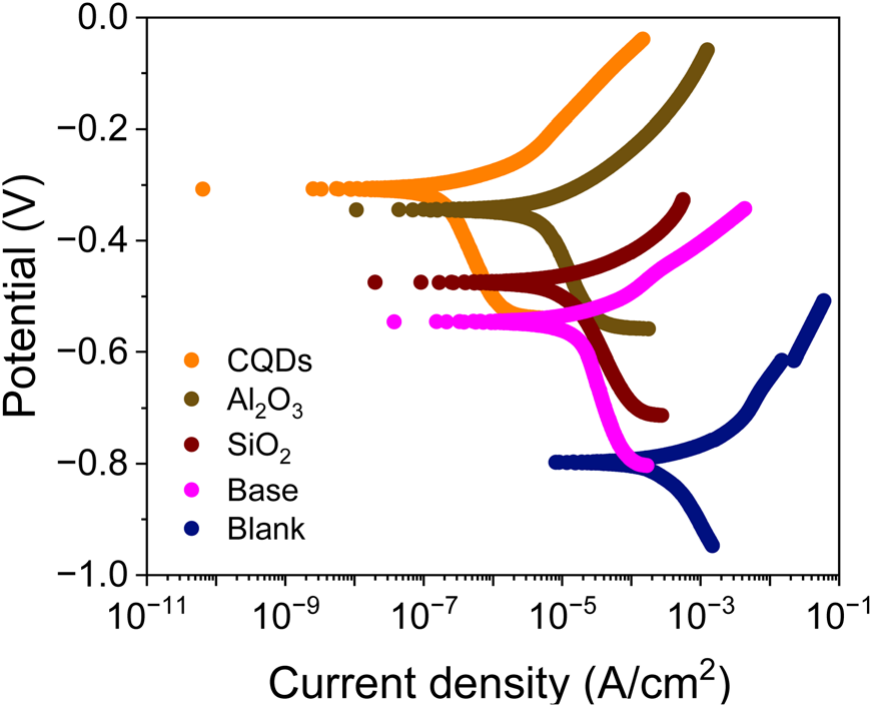
Polarization curves of the uncoated coupon (blank) and alkyd-urethane coating (base) with 100 mg L^−1^ of the nanoparticles evaluated in the brine saturated with CO_2_. The tests were carried out in a 3-electrode setup with reference (Ag/AgCl) and graphite as counter electrodes at 25 °C and atmospheric pressure.

Fig. 5 and Table 5 show the results of corrosion potential, corrosion current, the corrosion rate, and the corrosion inhibition efficiency of the coatings evaluated in CO_2_ saturated brine. Compared to the blank coupon, the base coating increases the corrosion potential from −0.78 V to −0.54 V and decreases the corrosion current from 5.062 × 10^−5^ A cm^−2^ to 0.218 × 1 A m^−2^. With respect to the base coating, the coatings with alumina, CQDs and silica increase the corrosion potential from −0.54 V of to −0.35, −0.31, and −0.47 V, respectively. Likewise, the corrosion current decreases from 0.218 × 10^−5^ A cm^−2^ to 0.067 × 10^−5^ A cm^−2^, 0.003 × 10^−5^ A cm^−2^, and 0.144 × 10^−5^ A cm^−2^, respectively. The corrosion rate of the blank was 4.72 × 10^−2^ mpy and it decreases to 0.0013 × 10^−2^ mpy with the base coating. With respect to the base, the coatings with alumina, CQDs and silica decrease the corrosion rate from 0.0013 × 10^−2^ mpy to 0.0002 × 10^−2^, 0.0001 × 10^−2^, and 0.0004 × 10^−2^ mpy, respectively. The corrosion inhibition efficiency for the base coating was 95.7%, while for the coatings with 100 mg L^−1^ of alumina, CQDs and silica nanoparticles were 98.7, 99.9, and 97.1%, respectively. This result indicates that the nanoparticles contribute to enhance efficiency by decreasing the rate of corrosion reaction in the metal.

**Table 5:** Electrochemical values obtained from the polarization test in CO_2_ saturated medium

	*E*_corr_ ± 0.01 (V)	*I*_corr_ (×10^−5^) ± 1 × 10^−9^ A cm^−2^	Corrosion rate (×10^−2^) ± 1 × 10^−5^ mpy	*η* (%) ± 0.1
Blank	−0.78	5.062	4.723	
Base	−0.54	0.218	0.0013	95.7
Base + Al_2_O_3_	−0.35	0.067	0.0002	98.7
Base + CQDs	−0.31	0.003	0.0001	99.9
Base + SiO_2_	−0.47	0.144	0.0004	97.1

Considering the previous results, the coating with CQDs exhibits the best performance in polarization test. This results could be explained by the fact that CQDs can adsorb carbon dioxide molecules on their surface because of the presence of functional groups such as hydroxyl, carboxyl, and amino groups.^122^ Specifically, CO_2_ dissolves in the brine to form carbonic acid (H_2_CO_3_) in CO_2_ saturated brine, which dissociates into bicarbonate (HCO_3_^−^) and hydrogen ions (H^+^).^123^ The formation of these products could be inhibited by the capture of CO_2_ molecules in the CQDs coating, reducing the availability of corrosive species.^124^ Similarly, silica nanoparticles can interact with CO_2_ and water molecules through physical adsorption, reducing the concentration of CO_2_ and water within the coating.^125^ In contrast, alumina nanoparticles are chemically inert and do not react with CO_2_. However, they can adsorb corrosive agents and prevent the metal surface corrosion.^126^ Compared to conventional coatings such as alkyd or polyurethane, alkyd-urethane coating offers superior synergistic effects with the nanoparticles evaluated. Author such us Mo *et al.*^127^ reported corrosion inhibition efficiencies of 79.15% with polyurethane coatings and 93.12% with the addition of graphene into the polymer matrix. The incorporation of nanoparticles like Al_2_O_3_, CQDs, and SiO_2_ in the alkyd-urethane matrix significantly reduces corrosion rates, increases electrochemical impedance, and enhances surface coverage due to the formation of a dense, crosslinked network. The nanoparticles promote stronger interfacial adhesion with both the polymer and the metal surface through hydrogen bonding and electrostatic interactions, leading to improved adhesion and reduced delamination. Additionally, the hybrid matrix offers environmental advantages than traditional epoxy systems due to the use of precursors obtained from renewable sources in the synthesis.

#### 3.1.4 Corrosion resistance test

**Fig. 6:**
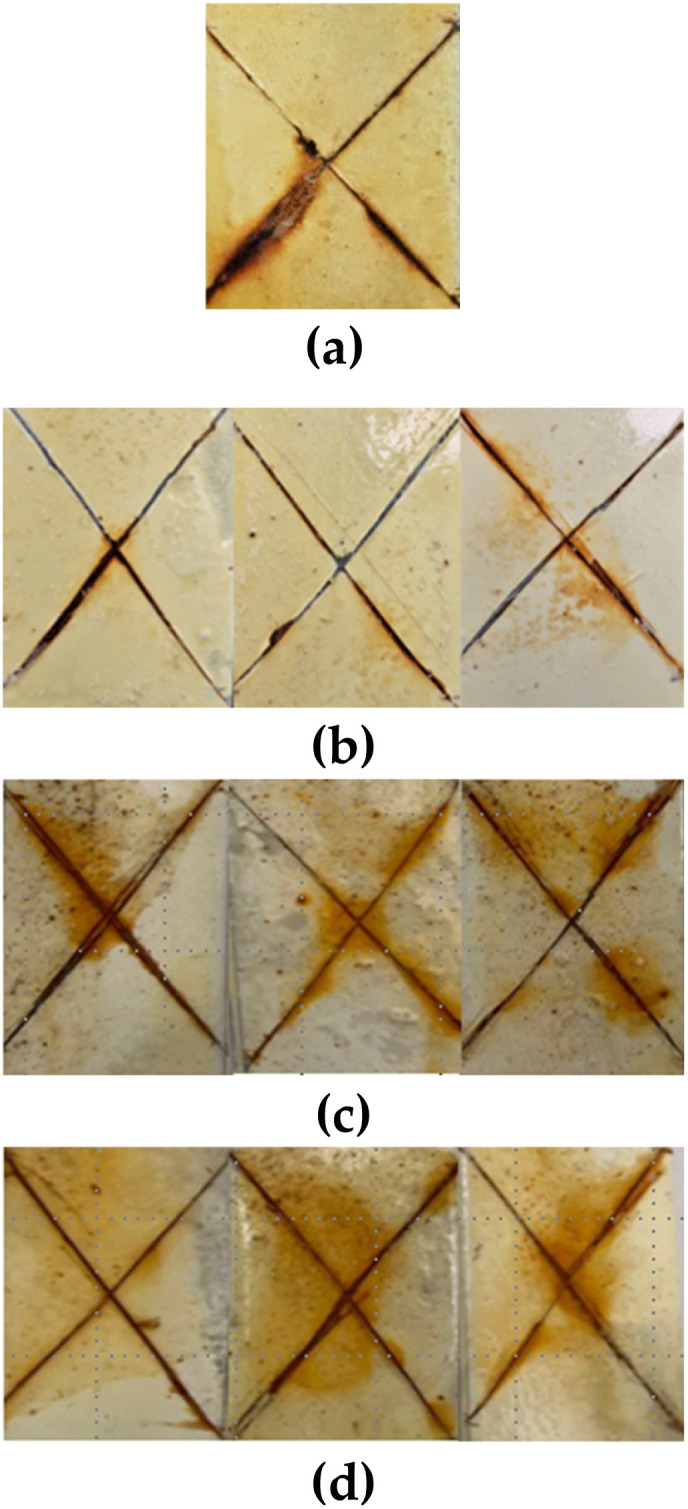
Images of the corrosion resistance test for coatings of (a) alkyd-urethane coating (base), (b) alumina, (c) CQDs, and (d) silica nanoparticles at concentrations of 10 100 and 1000 mg L^−1^, respectively.

The corrosion resistance test results for the evaluated coatings are shown in Fig. 6. It is possible to see signs of corrosion and coating detachment in the cut in Fig. 6a corresponding to the base coating. The coatings with alumina nanoparticles are shown in Fig. 6b. Alumina coatings show the slightest signs of corrosion, being 100 and 1000 mg L^−1^, the concentration with the best results. In Fig. 6c and d the coatings with CQDs and silica nanoparticles are shown. It is observed the presence of blistering and high levels of corrosion but without delamination. These results are in accordance with those reported by Kordzangeneh *et al.*^128^ who reported that alumina nanoparticles enhanced the anticorrosive performance of urethane coatings during corrosion resistance testing. Notably, the samples with 100 and 1000 mg L^−1^ of Al_2_O_3_ showed minimal corrosion and no delamination. These results are in accordance with the low corrosion currents values of 0.001 × 10^−5^ and 0.015 × 10^−5^ A cm^−2^ for 100, and 1000 mg L^−1^ of Al_2_O_3_. Furthermore, the inhibition efficiencies achieved are around 99.9%. This visual evidence supports the hypothesis that Al_2_O_3_ nanoparticles enhance the physical integrity and shielding capacity of the coating. Likewise, their ability to form hydrogen bonds with urethane groups and to reinforce the polymer matrix through interfacial interactions. This is in concordance with the results of the EIS test with a polarization decay of 44%. On the other hand, the coatings with CQDs at 100 mg L^−1^, showed excellent electrochemical behavior with the lowest corrosion current of 0.0009 × 10^−5^ A cm^−2^. However, the presence of surface blistering suggests that the hydrophilic nature of CQDs may contribute to localized water uptake, which can affect long-term stability as indicated by the EIS test with a 73.4% of polarization resistance decay.

Table 6 presents the results of the visual evaluation, including estimated corrosion creep, qualitative observations, and the corresponding D1654 ratings for each system. The base alkyd-urethane coating without nanoparticles exhibited visible corrosion products along the scribe line with moderate creep and undercutting estimated at approximately 1.5–2.0 mm. This corresponded to ASTM D1654 rating of 6–7, indicating limited protective capacity under aggressive saline conditions. In contrast, coatings modified with Al_2_O_3_ nanoparticles demonstrated the most effective corrosion resistance. At 100 mg L^−1^, the Al_2_O_3_-enhanced coating showed no visible corrosion or undercutting receiving a top rating of 10. While 10 and 1000 mg L^−1^ also performed well with minimal creep. These results may be attributed to the formation of dense, protective nanoparticle polymer networks and the barrier effect of Al_2_O_3_. Coatings containing CQDs exhibited moderate corrosion with blistering and undercutting observed in all concentrations. Ratings ranged from 6 to 7, with 100 mg L^−1^ showing slightly improved resistance compared to 10 and 1000 mg L^−1^. This behaviour could be due to the hydrophilicity of CQDs, which may enhance initial protection but promote localized water uptake and blistering over time. Similarly, coatings containing SiO_2_ nanoparticles showed moderate performance, with ratings between 6 and 8 depending on the concentration.

**Table 6:** Evaluation of corrosion degree in corrosion resistance test

Coating system	Nanoparticle concentration (mg L^−1^)	Visual observation	Estimated creep (mm)	Rating (0–10)
Base coating	—	Moderate corrosion, visible scribe rusting	∼1.5–2.0 mm	6–7
Al_2_O_3_	10	Minor rusting near scribe	≤0.5 mm	9
	100	No visible corrosion or blistering	≈0 mm	10
	1000	Minor rust near scribe	≤0.5 mm	9
CQDs	10	Blistering and rust along scribe	∼2.0 mm	6
	100	Blistering and some creep	∼1.5–2.0 mm	6–7
	1000	More corrosion than 100 mg L^−1^, slight blistering	∼2.0 mm	6
SiO_2_	10	Moderate rust, some scribe undercutting	∼1.0 mm	7–8
	100	Moderate corrosion, small creep	∼1.0–1.5 mm	7
	1000	Visible corrosion, slight blistering	∼1.5–2.0 mm	6–7

### 3.2 Rheological characterization

**Fig. 7:**
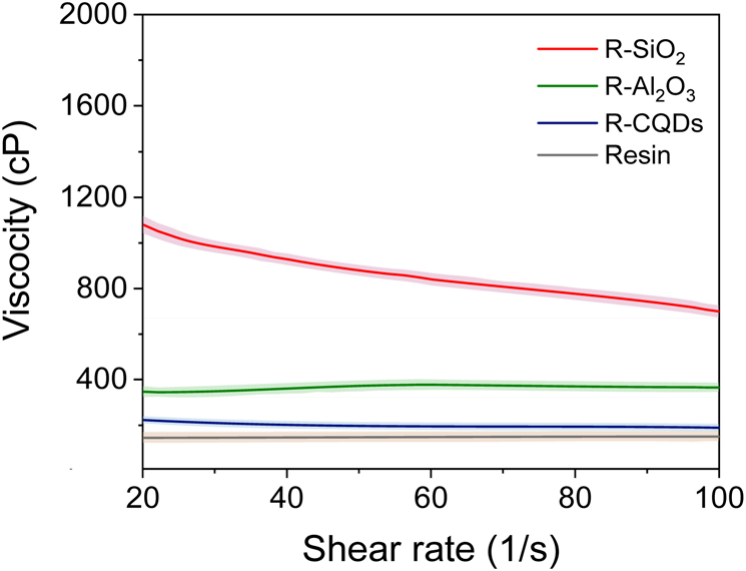
Effect of alumina, CQDs, and silica nanoparticles at 100 mg L^−1^ on the alkyd-urethane resin viscosity at 25 °C and atmospheric pressure. The test was performed at steady rheology conditions.

The effect of nanoparticles on coating viscosity is shown In Fig. 7. It can be observed that the resin and the resin with alumina exhibit Newtonian behavior. On the contrary, the resin with silica and CQDs nanoparticles at low shear rate show pseudoplastic behavior. The incorporation of nanoparticles can increase the viscosity of the base resin across a range of shear rates, suggesting that nanoparticles interact with the polymer matrix to form a more resistant network.^129–131^ Among the nanoparticles studied, silica generated the highest increase in viscosity, indicating a more significant interaction with the base resin or the formation of potentially larger aggregates. This behavior can be attributed to the formation of strong bonds between silanol and urethane groups.^132^ Otherwise, CQDs appear to have a less pronounced effect on viscosity. These results are consistent with those reported by Luo *et al.*,^133^ who obtained higher viscosity values at all evaluated shear rates with the addition of silica nanoparticles to a polyurethane coating. These findings are critical in terms of efficacy and functionality, including film formation, application methods, drying and curing. The increase in viscosity and the transition from Newtonian to pseudoplastic behavior can directly influence application processes such as spraying or brushing.^134,135^ For instance, the coating with SiO_2_ nanoparticles exhibits shear-thinning behavior that is advantageous for spray application.^136^ They allow for lower resistance to flow under shear while recovering viscosity quickly after deposition, reducing sagging and improving leveling.^136^ However, excessive viscosity may require adjustments in spraying equipment and increasing energy consumption prolonging curing times.^137^ In contrast, the Newtonian behavior observed in the base resin and the resin with Al_2_O_3_ offers more predictable and consistent flow properties, making the application process easier to control. Nonetheless, these systems may lack some of the performance benefits associated with pseudoplastic coatings, such as enhanced leveling or reduced sagging during application. The rheological characteristics of anticorrosive coatings are critical in terms of efficacy and functionality, including the film/coating formation, adhesion, application methods, drying and curing, durability, and environmental impact.

**Fig. 8:**
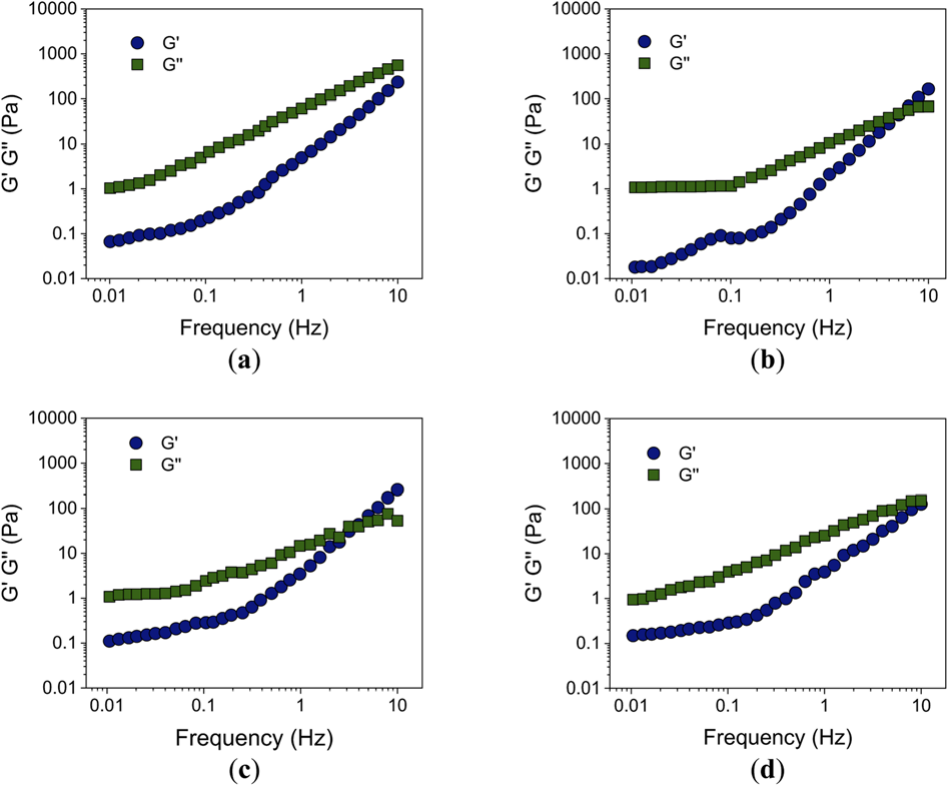
Frequency dependence of alkyd-urethane viscoelastic behavior. Storage modulus (*G*′) and loss modulus (*G*′′) as a function of frequency for (a) alkyd-urethane resin and with loads of 100 mg L^−1^ of (b) alumina (c) CQDs and (d) silica nanoparticles. The tests were carried out at 25 °C, atmospheric pressure, and dynamic rheology conditions. The linear viscoelastic region (LVR) was determined previously to the frequency sweep.

In Fig. 8, the effect of nanoparticles on the viscoelastic behavior of the alkyd-urethane resin is presented. The storage modulus (*G*′) and the loss modulus (*G*′′) of the alkyd-urethane resin are presented in Fig. 8a. As expected, the resin has a viscoelastic behavior. *G*′′ is generally greater than *G*′ across the measured frequency range. This indicates that the resin exhibits a more viscous or fluid-like behavior in this frequency range studied. It is observed that both *G*′ and *G*′′ increase with increasing frequency and that the slopes of both *G*′ and *G*′′ increase at higher frequencies. The effects of alumina, CQDs, and silica nanoparticles on the viscoelastic response are shown in Fig. 8b–d. The crossover points occurred at a lower frequency compared to the resin without nanoparticles, indicating an earlier transition from viscous to the elastic behavior.^96^. The increase in *G*′′ with increasing frequency indicates an increase in the elastic response of the resin. This suggests that the material stiffens and increases its elastic energy-storage capacity.^138^ The increase in the storage modulus across the frequency range indicates an improvement in the mechanical stability and rigidity of the alkyd-urethane resin.

Table 7 lists the thickness of the evaluated coatings. The addition of primers increased the thickness of the alkyd-urethane coating by approximately 45 μm. Conversely, the presence of nanoparticles had a small impact on the thickness of the coatings. The difference in coating thickness can be attributed to the application method.

**Table 7:** Thickness values of alkyd-urethane coating in the absence and the presence of nanoparticles at 100 mg L^−1^

Coupon	Thickness (μm)
Primer	45
Base	76
Base + primer	121
Base + Al_2_O_3_	125
Base + CQDs	131
Base + SiO_2_	116

### 3.3 SEM

**Fig. 9:**
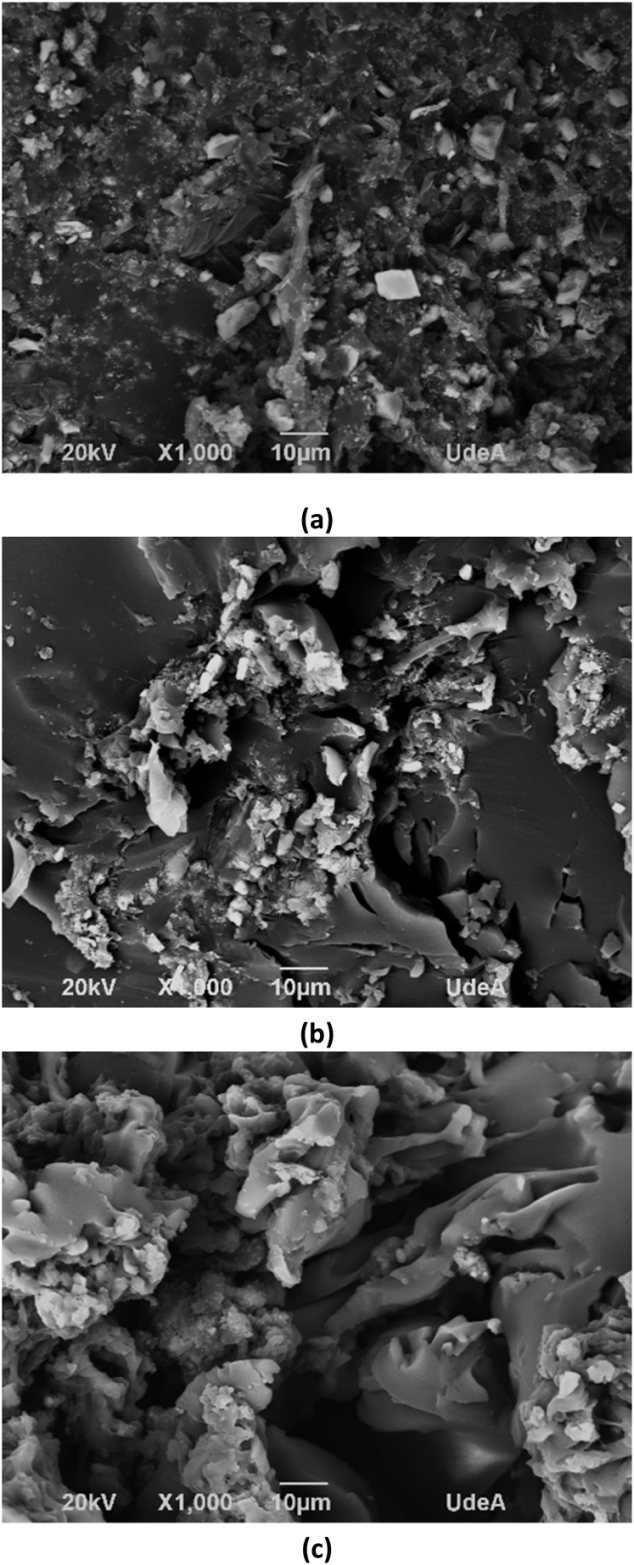
Micrographs of coatings obtained by SEM: (a) alkyd-urethane coating, (b) coating with 100 mg L^−1^ of alumina nanoparticles, and (c) coating with 100 mg L^−1^ of CQDs.

Considering the previous results, the coatings with alumina and CQDs were selected to evaluate the effect of nanoparticles in the microstructure of coating. Fig. 9 shows the SEM micrographs of coating in the absence and the presence of alumina, and CQDs nanoparticles at 100 mg L^−1^. The micrograph for the coating without nanomaterials reveals a heterogeneous and granular surface, with particles of varying sizes distributed relatively evenly. This suggests that the base has an irregular structure that can influence the interaction with the nanoparticles. The Al_2_O_3_ and CQDs nanoparticles were observed as white dots or areas of higher contrast. Specifically, the coating with CQDs (Fig. 9c) shows distinct agglomerates and a denser, rougher surface morphology, suggesting the presence of microdefects that could compromise its barrier properties and promote localized corrosion. The results indicate a stronger interaction between the Al_2_O_3_ nanoparticles and the polymer matrix, which contributes to the formation of a more compact and reinforced network that aligns with the improved corrosion resistance observed previously. Regarding the images analyses, aggregates of Al_2_O_3_ and CQDs have a mean diameter of 1.67 and 3.18 μm, respectively. The smaller diameter of aggregates of Al_2_O_3_ indicate higher dispersion in the alkyd-urethane matrix. Thus, the tortuosity increases, and the ion penetration is more difficult. Otherwise, the bigger size of CQDs into the coating can create paths and favors ion penetration. These results are in accordance with the EIS analysis where CQDs show higher polarization decays due to water uptake.

### 3.4 FTIR interactions analysis

**Fig. 10:**
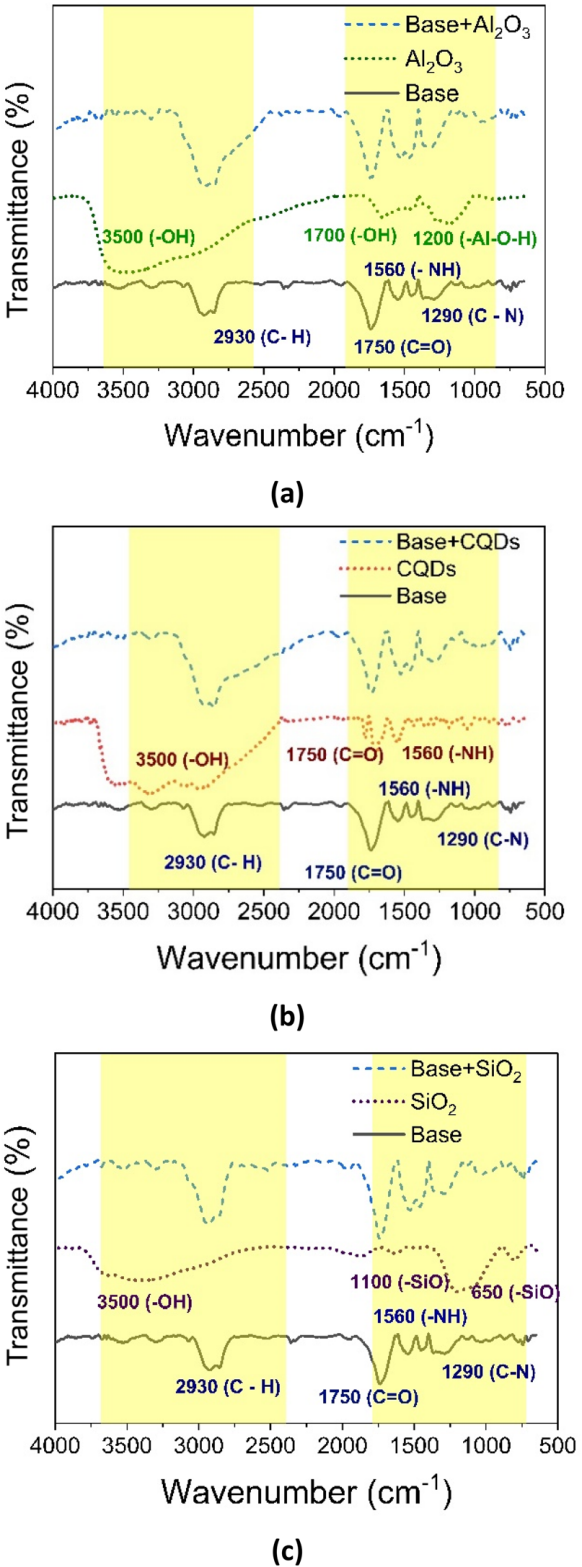
Evaluation of surface interactions to the alkyd-urethane coating and nanoparticles by FTIR: (a) alumina, (b) CQDs, and (c) silica nanoparticles.

The Fig. 10 shows the evaluation of surface interactions through FTIR to the alkyd-urethane coating and nanoparticles. Particularly, the Fig. 10a presents the FTIR spectra of alkyd-urethane coating, Al_2_O_3_ nanoparticles, and the blend alkyd-urethane and Al_2_O_3_ nanoparticles. For the FTIR spectra of alkyd-urethane coating, the characteristic band at 2930 cm^−1^ corresponds to aliphatic C–H stretching vibration. The strong absorption near 1750 cm^−1^ is associated to the stretching of carbonyl groups (C=O), typical of ester linkages in the alkyd-urethane resin. The peak at 1560 cm^−1^ is attributed to N–H bending vibrations of the urethane linkages while the C–N stretching appears at 1290 cm^−1^.^139^ The FTIR spectrum of Al_2_O_3_ is shown in Fig. 10a. The peak at 3500 cm^−1^ could be attributed to –OH stretching and the peak at 1700 cm^−1^ is assigned to Al–OH scissoring mode. The bands located in 1050, and 1250 cm^−1^ are attributed to the symmetrical deformation vibration and asymmetrical deformation vibration of Al–O–H modes.^140–142^ It is observed that the incorporation of Al_2_O_3_ nanoparticles in the alkyd-urethane resins affect the intensity of the peaks due to the fact that hydroxyl groups on the Al_2_O_3_ surface may interact with the functional groups present in the alkyd-urethane coating (carbonyl, urethane groups, and aliphatic chains).^143^ The FTIR spectra of alkyd-urethane coating, CQDs nanoparticles, and the blend alkyd-urethane coating and CQDs nanoparticles are depicted in Fig. 10b. The peaks at 3500 cm^−1^ (–OH), 1750 cm^−1^ (C=O), 1560 cm^−1^ (–NH), and 1049–1115 (–CO and –CN)^144^ of CQDs nanoparticles confirm the presence of functional groups that can interact with the functional groups of alkyd-urethane resin. When the CQDs are incorporated into the alkyd-urethane coating, the amplitude of the –OH (around 3000 cm^−1^) peak increases and the intensity of the peaks corresponding to the C=O and –NH is higher than the base FTIR spectrum. Again, this behaviour could relate to the surface interactions between functional groups. Fig. 10c illustrates the FTIR spectra of alkyd-urethane coating, SiO_2_ nanoparticles, and the blend alkyd-urethane coating and SiO_2_ nanoparticles. The spectrum of SiO_2_ nanoparticles reveals absorption bands attributed to Si–O bond flextion and to the asymmetric stretching of Si–O at 1000–1250 cm^−1^ and the band of 3500 cm^−1^ correspond to the –OH stretching of the silanol groups.^84,145^ Regarding the blend FTIR spectrum is observed a slightly increase in the intensity of the peak at 2930 cm^−1^ and the peaks in the region from 700 to 1100 cm^−1^ compared with the base. This result suggests interactions between the SiO_2_ nanoparticles and the alkyd-urethane coating (polymer). It is important to note that the changes in the peak intensity were more significant to the blends based on alumina and CQDs nanoparticles. Overall, the FTIR analysis demonstrates that the incorporation of nanoparticles does not significantly alter the chemical structure of the base coating. However, some changes in the intensity of the peaks are observed. These results confirm and indicate interfacial and surface interactions that may enhance the coatings stability and barrier performance. Furthermore, the results are in accordance with the results obtained from EIS tests, which indicated the best electrochemical performance for the coating based on Al_2_O_3_, showing the higher changes in the spectral bands.

## 4. Conclusions

The incorporation of alumina, CQDs, and silica nanoparticles into an alkyd-urethane-based coating was evaluated to enhance the corrosion resistance of well pipelines exposed to brine and CO_2_-containing brine. Polarization tests demonstrated that all nanoparticle-modified coatings exhibited a less negative corrosion potential, and a lower corrosion current compared to the base coating. Among the coatings studied, those containing 100 mg L^−1^ of CQDs showed the least negative potential and the lowest corrosion current with a potential shift from −0.60 to −0.28 V and current density decreased from 0.088 × 10^−5^ to 0.0009 × 10^−5^ compared to the base coating. This result suggests that CQDs provide the greatest electrochemical stability and effectively reduce the propensity for corrosion initiation. However, electrochemical impedance spectroscopy results indicated that the Al_2_O_3_-based coating showed the highest polarization resistance over time with values of 23.1 kΩ and the lowest decay in 20 days of exposure to brine with 44.6%. This trend implies that alumina nanoparticles significantly enhance the barrier properties of the coating, minimizing electrolyte penetration and charge transfer processes. Regarding rheological behaviour, the addition of nanoparticles increased the viscosity. CQDs had the least impact on the viscosity whereas the incorporation of alumina and silica nanoparticles had a higher impact. Furthermore, the gelation point decreased, improving the cure time of resin in the pipelines.

The results of polarization, impedance, corrosion resistance, and rheological tests showed that the surface interaction of functional groups between nanoparticles and polymers has a significant influence on the final corrosion coating protection. The main differences are associated with the chemical structure, the size, and the *z*-potential of nanoparticles and alkyd-urethane resin. The use of nanoparticles as an additive in coating based on castor oil is a promising alternative because of their environmentally friendly nature and technical performance. The corrosion resistance and mechanical durability make these coatings suitable for protecting pipelines, storage tanks, and equipment in the broader oil and gas sector, where exposure to harsh chemical conditions is common. Additionally, the performance in saline environments suggests potential use in marine applications where corrosion is a significant challenge. For future research, long-term durability studies under thermal and chemical stresses are recommended to validate performance in field application. Furthermore, spraying techniques for industrial scale-up should be evaluated economic analysis should be considered to determine the coating application in a well pipelines field oil. However, specialized studies for application in the well pipelines of the oil and gas industries should be required and are subject to future communications.

## Author contributions

J. D. Q.: conceptualization; data curation; formal analysis; investigation; methodology; software; validation and writing – original draft preparation. Y. V.: conceptualization; data curation; formal analysis; investigation; methodology; validation and writing – original draft preparation. H. I.: methodology, formal analysis, conceptualization. C. G.: methodology, formal analysis, conceptualization. E. A. T.: methodology, formal analysis, conceptualization. L. R.: conceptualization; formal analysis; and writing—reviewing and editing. C. A. F: conceptualization; formal analysis; and writing—reviewing and editing F. B. C.: conceptualization; formal analysis; and writing—reviewing and editing. All authors have read and agreed to the published version of the manuscript.

## Conflicts of interest

The authors declare that they have no known competing financial interests or personal relationships that could have appeared to influence the work reported in this paper.

## Data Availability

The authors declare that all the data in this manuscript are available upon request.

## References

[cit1] Perez T. E. (2013). JOM.

[cit2] Olajire A. A. (2017). J. Mol. Liq..

[cit3] Simons M. R. , Report of Offshore Technology Conference (OTC) Presentation, NACE Interantional Oil and Gas Production, 2008

[cit4] Brondel R. D. E., Hayman A., Hill D., Mehta S., Semerad T. (1987). J. Pet. Technol..

[cit5] Mubarak G., Verma C., Barsoum I., Alfantazi A., Rhee K. Y. (2023). J. Taiwan Inst. Chem. Eng..

[cit6] Wu H., Zhao H., Li X., Feng X., Chen Y. (2022). Ocean Eng..

[cit7] Askari M., Aliofkhazraei M., Jafari R., Hamghalam P., Hajizadeh A. (2021). Appl. Surf. Sci. Adv..

[cit8] Popoola L., Grema A., Latinwo G., Gutti B., Balogun A. (2013). Int. J. Ind. Chem..

[cit9] Speight V. L. (2014). Procedia Eng..

[cit10] Nind T. E. W. , Fundamentos de producción y mantenimiento de pozos petroleros, Limusa, 1987

[cit11] Alfonso Alarcón M. A. and Castañeda A. F., Evaluación de la viabilidad técnico-financiera de las operaciones de Workover mediante simulación numérica para la reapertura del campo Hato nuevo, Bachelor thesis, Fundación Universidad de América, 2017

[cit12] Aslam R., Mobin M., Zehra S., Aslam J. (2022). J. Mol. Liq..

[cit13] Sridhar N., Thodla R., Gui F., Cao L., Anderko A. (2018). Corros. Eng., Sci. Technol..

[cit14] Mirza M., Rasu E., Desilva A. (2016). Am. Chem. Sci. J..

[cit15] Al-Janabi Y. T. , An Overview of Corrosion in Oil and Gas Industry: Upstream, Midstream, and Downstream Sectors, 2020

[cit16] Sanni O., Iwarere S. A., Daramola M. O. (2023). Sustainability.

[cit17] Tamilselvi B., Bhuvaneshwari D. S., Karuppasamy P., Padmavathy S., Nikhil S., Siddegowda S. B., Ananda Murthy H. C. (2024). ACS Phys. Chem. Au.

[cit18] Ragu M., Karuppasamy P., Thirupathi J., Ganesan M., Rajendran T., Sivasubramanian V. K. (2022). Asian J. Chem..

[cit19] Baluchamy T., Bhuvaneshwari D. S., Padmavathy S., Rajapandian V., Karuppasamy P. (2024). Prot. Met. Phys. Chem. Surf..

[cit20] Wang Q., Wang R., Zhang Q., Zhao C., Zhou X., Zheng H., Zhang R., Sun Y., Yan Z. (2023). Molecules.

[cit21] Bendaif H. (2016). et al.. J. Mater. Environ. Sci..

[cit22] Kemel M. (2024). J. Ind. Eng. Chem..

[cit23] Jin Z. H., Ge H. H., Lin W. W., Zong Y. W., Liu S. J., Shi J. M. (2014). Appl. Surf. Sci..

[cit24] Lochyński P., Domańska M., Dziedzic R., Hamal K. (2023). Materials.

[cit25] Wang J., Ryan D., Anthony E. J., Wildgust N., Aiken T. (2011). Energy Procedia.

[cit26] Varela F., Tan M. Y. J., Forsyth M. (2015). Electrochim. Acta.

[cit27] Yang G.-J. and Li G.-R., in Advanced Nanomaterials and Coatings by Thermal Spray, Elsevier, 2019, pp. 257–289, 10.1016/B978-0-12-813870-0.00008-5

[cit28] Caramitu A. R., Ciobanu R. C., Lungu M. V., Lungulescu E.-M., Scheiner C. M., Aradoaei M., Bors A. M., Rus T. (2024). Polymers.

[cit29] Attarzadeh N., Molaei M., Babaei K., Fattah-alhosseini A. (2021). Surf. Interfaces.

[cit30] Ren B., Chen Y., Li Y., Li W., Gao S., Li H., Cao R. (2020). Chem. Eng. J..

[cit31] Idumah C. I., Obele C. M., Emmanuel E. O., Hassan A. (2020). Surf. Interfaces.

[cit32] Lathabai S., Ottmüller M., Fernandez I. (1998). Wear.

[cit33] Lauer R. S. and Tuboscope N., SPE/IADC-173006-MS Historical Advances in Drill Pipe Internal Coating Systems and the Performance of Liquid versus Powder Applied Coating Systems, 2015

[cit34] Lu C. , Feng C., Zhu L., Jiang L., Gao G., Han L. and Feng Y., Advances in Materials Processing, 2018, pp. 1075–1082, 10.1007/978-981-13-0107-0_102

[cit35] Lauer R. S. , SPE-162182-MS Advancements in the abrasion resistance of internal plastic coatings, paper presented at the Abu Dhabi International Petroleum Conference and Exhibition, Abu Dhabi, UAE, 2012

[cit36] Chen E. Y. and Ahmed T., SPE 49211 Why Internally Coated Piping Is Used for the Worldrs Largest Seawater Injection System, 1998

[cit37] Nunn J. A. (1987). Oil Gas J..

[cit38] Fitzgerald A. , Groves S., Gosch S., Morey S., David P., Ii P., Piskurich N. and Sathuvalli U. B., SPE 115313 Alternatives to 25% Chrome for Water-Injection Tubing in Deep Water, 2008

[cit39] Ogbonna V. E., Popoola A. P. I., Popoola O. M., Adeosun S. O. (2022). Polym. Bull..

[cit40] Klimchuk S., Shang M., Samuel M. S., Niu J. (2020). ACS Appl. Mater. Interfaces.

[cit41] Heflin D. G., Mansson J.-A. E. (2022). Polym. Polym. Compos..

[cit42] Khatoon H., Ahmad S. (2017). J. Ind. Eng. Chem..

[cit43] Samimi A., Zarinabadi S., Kootenaei A., Azimi A., Mirzaei M. (2020). Chem. Methodol..

[cit44] Peng Y.-J., He X., Wu Q., Sun P.-C., Wang C.-J., Liu X.-Z. (2018). Polymer.

[cit45] Farh H. M. H., Ben Seghier M. E. A., Zayed T. (2023). Eng. Fail. Anal..

[cit46] International Society of O. and Polar E., The Proceedings of the Seventeenth (2007) International Offshore and Polar Engineering Conference : Lisbon, Portugal, July 1-6, 2007, International Society of Offshore and Polar Engineers, 2007

[cit47] Sreehari H., Sethulekshmi A. S., Saritha A. (2022). Macromol. Mater. Eng..

[cit48] Zhang S. Y., Kong Y., Zhang Z. S., Zhang X. Y. (2003). J. Appl. Electrochem..

[cit49] Das S., Pandey P., Mohanty S., Nayak S. K. (2017). Polym.-Plast. Technol. Eng..

[cit50] Cunningham M. F., Campbell J. D., Fu Z., Bohling J., Leroux J. G., Mabee W., Robert T. (2019). Green Chem..

[cit51] Nardeli J. V., Fugivara C. S., Taryba M., Montemor M. F., Ribeiro S. J. L., Benedetti A. V. (2020). Corros. Sci..

[cit52] Bhat S. I., Ahmad S. (2018). Prog. Org. Coat..

[cit53] Patil A. M., Jagtap R. N. (2021). J. Coat. Technol. Res..

[cit54] Patel V. R., Dumancas G. G., Viswanath L. C. K., Maples R., Subong B. J. J. (2016). Lipid Insights.

[cit55] Zhe Z., Yuxiu A. (2018). Nanotechnol. Rev..

[cit56] Esmaeili A., Patel R. B., Singh B. P. (2011). AIP Conf. Proc..

[cit57] Peng B., Tang J., Luo J., Wang P., Ding B., Tam K. C. (2018). Can. J. Chem. Eng..

[cit58] Rao C. N. R., Cheetham A. K. (2001). J. Mater. Chem..

[cit59] Saji V. S. , in Corrosion Protection and Control Using Nanomaterials, Elsevier, 2012, DOI: 10.1533/9780857095800.1.3, pp. 3–15

[cit60] Edelstein A. S., Murday J. S., Rath B. B. (1997). Prog. Mater Sci..

[cit61] Bahramnia H., Mohammadian Semnani H., Habibolahzadeh A., Abdoos H. (2020). J. Compos. Mater..

[cit62] Niroumandrad S., Rostami M., Ramezanzadeh B. (2016). Prog. Org. Coat..

[cit63] Thiruvoth D. D., Ananthkumar M. (2022). Mater. Today: Proc..

[cit64] Jena K. K., Narayan R., Raju K. V. S. N. (2015). Prog. Org. Coat..

[cit65] El Saeed A. M., Abd El-Fattah M., Azzam A. M., Dardir M. M., Bader M. M. (2016). Int. J. Biol. Macromol..

[cit66] Sekhavat Pour Z., Ghaemy M., Bordbar S., Karimi-Maleh H. (2018). Prog. Org. Coat..

[cit67] Zahra M., Ullah H., Javed M., Iqbal S., Ali J., Alrbyawi H., Samia, Alwadai N., Ibrahim Basha B., Waseem A., Sarfraz S., Amjad A., Awwad N. S., Ibrahium H. A., Somaily H. H. (2022). Inorg. Chem. Commun..

[cit68] Jiang C.-C., Cao Y.-K., Xiao G.-Y., Zhu R.-F., Lu Y.-P. (2017). RSC Adv..

[cit69] Saji V. S. , Corrosion Protection at the Nanoscale, Elsevier, 2020

[cit70] Rohani R., Dzulkharnien N. S. F., Harun N. H., Ilias I. A. (2022). Bioinorg. Chem. Appl..

[cit71] Shirehjini F. T., Danaee I., Eskandari H., Zarei D. (2016). J. Mater. Sci. Technol..

[cit72] Janaki G. B., Xavier J. R. (2021). Surf. Coat. Technol..

[cit73] Chang X., Chen X., Zhang Q., Lei Y., Wang D., Li J., Sun S. (2021). Corros. Commun..

[cit74] Salzano de Luna M. (2022). Adv. Mater. Interfaces.

[cit75] Ifijen I. H., Maliki M., Odiachi I. J., Aghedo O. N., Ohiocheoya E. B. (2022). Chem. Afr..

[cit76] Samardžija M., Alar V., Špada V., Stojanović I. (2022). Coatings.

[cit77] Janaki G. B., Xavier J. R. (2021). Surf. Coat. Technol..

[cit78] Zeng Q., Yin H., Li Q., Bulyk I. I., Wang Z., Zhou S. (2025). Surf. Coat. Technol..

[cit79] Nunes R. d. S., Magno Paiva V., de Oliveira S. M., da Silva de Almeida C. M., de Oliveira M. S., de Araujo J. R., Archanjo B. S., Suguihiro N. M., D'Elia E. (2024). ACS Omega.

[cit80] Atazadeh N., Nogorani F. S. (2024). Mater. Corros..

[cit81] Udoh I. I., Ekerenam O. O., Daniel E. F., Ikeuba A. I., Njoku D. I., Kolawole S. K., Etim I.-I. N., Emori W., Njoku C. N., Etim I. P., Uzoma P. C. (2024). Adv. Colloid Interface Sci..

[cit82] Franco C. A., Candela C. H., Gallego J., Marin J., Patiño L. E., Ospina N., Patiño E., Molano M., Villamil F., Bernal K. M., Lopera S. H., Franco C. A., Cortés F. B. (2020). Ind. Eng. Chem. Res..

[cit83] Franco C. A., Nassar N. N., Cortés F. B. (2014). J. Colloid Interface Sci..

[cit84] Montes D., Henao J., Taborda E. A., Gallego J., Cortés F. B., Franco C. A. (2020). ACS Omega.

[cit85] Villada Y., Inciarte H., Gomez C., Cardona S., Orozco L. M., Estenoz D., Rios L. (2023). Prog. Org. Coat..

[cit86] ASTM G59-23 , Standard Test Method for Conducting Potentiodynamic Polarization Resistance Measurements, 2023, pp. 237–239, 10.1520/G0059-23

[cit87] Scully J. R. and Kelly R. G., Methods for Determining Aqueous Corrosion Reaction Rates, Corrosion: Fundamentals, Testing, and Protection, ASM Handbook, ed. S. D. Cramer and B. S. Covino Jr, ASM International, 2003, vol. 13A, pp. 68–86

[cit88] Leeds S. and Leeds J., in Oil and Gas Pipelines, 2015, pp. 457–484, 10.1002/9781119019213.ch32

[cit89] Zafar S., Kahraman R., Shakoor R. A. (2024). Eur. Polym. J..

[cit90] He H., Shen X., Nie Z. (2023). Prog. Polym. Sci..

[cit91] Rance G. A., Marsh D. H., Bourne S. J., Reade T. J., Khlobystov A. N. (2010). ACS Nano.

[cit92] Hetemi D., Pinson J. (2017). Chem. Soc. Rev..

[cit93] Jackson A. W., Fulton D. A. (2013). Polym. Chem..

[cit94] Kao J., Thorkelsson K., Bai P., Rancatore B. J., Xu T. (2013). Chem. Soc. Rev..

[cit95] Hong R. Y. and Chen Q., Organic-Inorganic Hybrid Nanomaterials, 2014, pp. 1–38, 10.1007/12_2014_286

[cit96] Chen Y., Zhou S., Yang H., Gu G., Wu L. (2004). J. Colloid Interface Sci..

[cit97] Wang G., Wang R., Wang C., Tang C., Zhang F. (2023). Int. J. Mech. Sci..

[cit98] Vishvanathperumal S., Kannan A. (2025). Colloid Polym. Sci..

[cit99] Ding C., Liu L., Feng L. (2023). AIP Adv..

[cit100] Madhi A., Shirkavand Hadavand B. (2022). J. Compos. Mater..

[cit101] Guzmán J. D., Betancur S., Carrasco-Marín F., Franco C. A., Nassar N. N., Cortés F. B. (2016). Energy Fuels.

[cit102] Dandan Doganci M., Sevinç H. (2023). ACS Omega.

[cit103] Hosseinpour A., Rezaei Abadchi M., Mirzaee M., Ahmadi Tabar F., Ramezanzadeh B. (2021). Surf. Interfaces.

[cit104] Sharma K., Hooda A., Goyat M. S., Rai R., Mittal A. (2022). Ceram. Int..

[cit105] Bahramnia H., Semnani H. M., Habibolahzadeh A., Abdoos H. (2021). Surf. Coat. Technol..

[cit106] Latif Z., Shahid K., Anwer H., Shahid R., Ali M., Lee K. H., Alshareef M. (2024). Nanoscale.

[cit107] Ru Y., Waterhouse G. I. N., Lu S. (2022). Aggregate.

[cit108] John V. L., Nair Y., Vinod T. P. (2021). Part. Part. Syst. Charact..

[cit109] Nejad M., Cooper P., Landry V., Blanchet P., Koubaa A. (2015). Prog. Org. Coat..

[cit110] Gilak Hakimabadi S., Ehsani M., Esfandeh M. (2024). Polym.-Plast. Technol. Mater..

[cit111] Xu L.-Y., Wang X.-Y., Lin Y.-Z., Huang Y., Tao C.-C., Zhang D.-W. (2025). Chin. J. Polym. Sci..

[cit112] Santos B. P. S., Arias J. J. R., Jorge F. E., Santos R. É. P. D., Fernandes B. S., Candido L. S., Peres A. C. C., Chaves É. G., Marques M. F. V. (2021). Mater. Today Commun..

[cit113] Li D., Chen Q., Chun J., Fichthorn K., De Yoreo J., Zheng H. (2023). Chem. Rev..

[cit114] Perera D. Y., Selier P. (1973). Prog. Org. Coat..

[cit115] Wool R. P. , Self-Healing Materials, Wiley, 2008 10.1039/b711716g 32907199

[cit116] van der Wel G. K., Adan O. C. G. (1999). Prog. Org. Coat..

[cit117] Castela A. S., Simões A. M. (2003). Corros. Sci..

[cit118] Dolatzadeh F., Jalili M. M., Moradian S. (2013). Mater. Corros..

[cit119] Hensen E. J. M., Poduval D. G., Degirmenci V., Ligthart D. A. J. M., Chen W., Maugé F., Rigutto M. S., Veen J. A. R. v. (2012). J. Phys. Chem. C.

[cit120] Markowitz M., Schoen P., Kust P., Gaber B. (1999). Colloids Surf., A.

[cit121] Lu Y., Huang L., Guo Y., Yang X. (2021). Carbon.

[cit122] Liu Z., Hou W., Guo H., Wang Z., Wang L., Wu M. (2023). ACS Appl. Mater. Interfaces.

[cit123] Mathiazhagan A., Joseph R. (2011). Int. J. Chem. Eng. Appl..

[cit124] Zhao F., Cui C., Dong S., Xu X., Liu H. (2023). Sep. Purif. Technol..

[cit125] Sattari A., Ramazani A., Aghahosseini H., Aroua M. K. (2021). J. CO_2_ Util..

[cit126] Arun K. L., Udhayakumar M., Radhika N. (2023). J. Bio- Tribo-Corros..

[cit127] Mo M., Zhao W., Chen Z., Liu E., Xue Q. (2016). RSC Adv..

[cit128] Kordzangeneh S., Naghibi S., Esmaeili H. (2018). J. Mater. Eng. Perform..

[cit129] Vassileva E., Friedrich K. (2003). J. Appl. Polym. Sci..

[cit130] Exner W., Arlt C., Mahrholz T., Riedel U., Sinapius M. (2012). Compos. Sci. Technol..

[cit131] Guo Z., Pereira T., Choi O., Wang Y., Hahn H. T. (2006). J. Mater. Chem..

[cit132] Chen G., Zhou S., Gu G., Yang H., Wu L. (2005). J. Colloid Interface Sci..

[cit133] Luo Z., Hong R. Y., Xie H. D., Feng W. G. (2012). Powder Technol..

[cit134] Altay B. N. and Lewis C. L., in Standardized Procedures and Protocols for Starch, ed. S. Punia Bangar, Springer US, New York, NY, 2024, pp. 145–196, 10.1007/978-1-0716-3866-8_6

[cit135] Patti A., Acierno D. (2023). J. Vinyl Add. Tech..

[cit136] Band C., Merin A. P., Srinivasan V. (2024). Int. J. Heat Fluid Flow.

[cit137] Pendar M.-R., Rodrigues F., Páscoa J. C., Lima R. (2022). Phys. Fluids.

[cit138] Amorim A. A. P. O., Oliveira M. G., Mancini M. C., Sirqueira A. S. (2021). SN Appl. Sci..

[cit139] Villada Y., Inciarte H., Gomez C., Cardona S., Orozco L. M., Estenoz D., Rios L. (2023). Prog. Org. Coat..

[cit140] Amirsalari A., Farjami Shayesteh S. (2015). Superlattices Microstruct..

[cit141] Xu N., Liu Z., Bian S., Dong Y., Li W. (2016). Ceram. Int..

[cit142] Bin Mokaizh A. A., Shariffuddin J. H., Baarimah A. O., Al-Fakih A., Mohamed A., Baarimah S. O., Al-Mekhlafi A.-B. A., Alenezi H., Olalere O. A., Saeed A. A. (2022). Materials.

[cit143] Ameri E., Sadeghi M., Zarei N., Pournaghshband A. (2015). J. Membr. Sci..

[cit144] Rosales S., Zapata K., Medina O. E., Rojano B. A., Taborda E. A., Cortés F. B., Pérez-Cadenas A. F., Bailón-García E., Carrasco-Marín F., Franco C. A. (2025). Nanoscale Adv..

[cit145] Metin C. O., Lake L. W., Miranda C. R., Nguyen Q. P. (2011). J. Nanopart. Res..

